# Changes in Depressive Symptoms, Perceived Stress, and Food Security Among Study Participants With Metabolic Syndrome During a COVID-19–Mandated Research Pause

**DOI:** 10.5888/pcd19.220206

**Published:** 2022-12-29

**Authors:** Barbara Lohse, Anahi Ramirez, Jenna Hickey, Lisa Bailey-Davis, Betty Drees, Kevin S. Masters, Elizabeth H. Ruder, Nicole Trabold

**Affiliations:** 1 Wegmans School of Health and Nutrition, Rochester Institute of Technology, Rochester, New York; 2 Department of Psychology, University of Missouri–Kansas City, Missouri; 3 Department of Population Health Sciences, Geisinger Health, Danville, Pennsylvania; 4 Department of Biomedical and Health Informatics, University of Missouri–Kansas City, Missouri; 5 Graduate School of the Stowers Institute for Medical Research, Kansas City, Missouri; 6 Department of Psychology and Anschutz Health and Wellness Center, University of Colorado Denver, Denver, Colorado

## Abstract

**Introduction:**

We explored how depressive symptoms, perceived stress, and food security of people with metabolic syndrome (MetS) changed during the COVID-19 pandemic.

**Methods:**

An online survey was administered from October 2019 through March 2020, to participants in a 2-year lifestyle intervention trial to reverse MetS; the survey was repeated during the COVID-19 pandemic. Outcomes were a change in depressive symptoms, perceived stress, and food security as measured by the Patient Health Questionnaire-8 (PHQ-8), Perceived Stress Scale, and US Department of Agriculture’s 10-item Adult Food Security Module. We analyzed changes in outcomes with measures of association, paired *t* tests, repeated measures, and independent *t* tests.

**Results:**

Survey respondents (N = 132) were mostly female (67%), White (70%), and middle-aged, with a median income of $86,000. Frequency of depressive symptoms increased from baseline to follow-up and the increase was related to lower mean (SD) baseline vitality (44.4 [20.7] vs 60.3 [18.9]; *P* = .01) and mental health decline (71.0 [14.3] vs 82.0 [10.4]; *P* = .002). Mean (SD) perceived stress was significantly higher at baseline than follow-up (18.5 [6.4] vs 14.9 [7.2]; *P* < .001). Food security increased from 83% at baseline to 90% at follow-up (*P* < .001). Movement to or continued food insecurity (n = 13) tended to be associated with a racial or ethnic minority group (*P* = .05).

**Conclusion:**

A sample at high risk for COVID-19 did not experience increased stress or food insecurity, but demonstrated increased depressive symptoms after the onset of the COVID-19 pandemic, with some baseline susceptibility.

**Summary**

**What is already known on this topic?**

People with metabolic syndrome (MetS) are at higher-than-normal risk for severe COVID-19 infection and its complications.

**What is added by this report?**

Highly educated and economically secure people with MetS reported less stress and greater food security during the pandemic but experienced more depressive symptoms, compared with a prepandemic baseline. Prepandemic vitality and mental health status contributed to psychological response among people with MetS.

**What are the implications for public health practice?**

Screening and assessing people with chronic diseases such as MetS for the psychosocial sequelae of the pandemic would benefit public health, even among people with personal, educational, and financial resources.

## Introduction

The COVID-19 pandemic has provided unprecedented challenges to the health and well-being of people worldwide, including food insecurity (1) and psychosocial stressors affecting depressive symptoms and mental health (2). People with metabolic syndrome (MetS) are at higher-than-normal risk for severe COVID-19 infection and its complications (3). MetS is a condition that consists of 3 or more of the following 5 risk factors: abdominal adiposity, elevated triglyceride levels, low high-density lipoprotein (HDL) cholesterol, high blood pressure, and insulin resistance (4). Risk of food insecurity (5), depressive symptoms, and poor mental health–related quality of life (6) is greater among people with MetS than among people without this condition. Therefore, the effect of the pandemic on food security, stress, depression, and eating behavior among people with MetS is of concern.

The public health mandate to socially isolate to prevent the spread of COVID-19 required a pause in the Enhanced Lifestyle for Metabolic Syndrome (ELM) trial (elmtrial.org). ELM is a multisite (Wilkes Barre and Danville, Pennsylvania; Rochester, New York; Chicago, Illinois; Denver, Colorado; Kansas City, Missouri) randomized clinical trial investigating the efficacy of an in-person, group-based intervention compared with a self-directed intervention to reverse MetS. The public health mandate occurred after the first of 4 cohorts was recruited and an in-person group-based intervention had just started for 1 site. Outcome assessments from intervention studies involving people with MetS that were underway before the pandemic may be vulnerable to the unexpected events stemming from pandemic-related public health recommendations (eg, stay-at-home orders, wearing face masks, social distancing) and a pause in any treatment arm, and may also affect future recruitment interest. The objective of this study was to examine changes in depressive symptoms, perceived stress, and food security among ELM study participants during the COVID-19–mandated research pause.

## Methods

This study involved data collected at baseline (October 16, 2019–March 12, 2020) and 3 to 9 months later (June 4, 2020–July 28, 2020) during a COVID-19–mandated trial pause in the 5-site ELM trial (ClinicalTrials.gov identifier: NCT04036006). Trial enrollment at each of the 5 sites was conducted in 4 cohort waves; this study consists of cohort 1 data from all 5 sites. The trial pause occurred before any intervention at 4 of the 5 sites. One site had completed 2 group meetings, and at this site and another site, participants who were randomized to the self-directed arm continued to receive evidence-based information related to health and well-being by mail every month, and telephone calls every 3 months.

### Participant recruitment

Trial recruitment was unique to each clinical site but generally included 3 strategies: 1) identification of potential participants through electronic health records, 2) referral by a medical provider, and 3) self-referral. Inclusion and exclusion criteria ensured that participants did not have a diagnosis of diabetes, stroke, heart disease, or related high-risk conditions; mental health illnesses including eating disorders; mobility limitations; history of bariatric surgery; or use of medications that could affect weight, appetite, or eating behavior. Eligible people had a diagnosis of MetS as defined by having at least 3 of 5 indicators: 1) waist circumference of ≥102 cm (40.2 inches, men) and ≥88 cm (34.6 inches, women), 2) fasting blood glucose 100 to 125 mg/dL (inclusive) or taking metformin, 3) systolic blood pressure ≥130 mm Hg, diastolic blood pressure ≥85 mm Hg, or treatment with antihypertensive medication, 4) fasting triglycerides ≥150 mg/dL or treatment of hypertriglyceridemia; 5) HDL cholesterol <40 mg/dL (men) or <50 mg/dL (women) or treatment of low HDL cholesterol.

Informed consent documents, approved by the Rush University Institutional Review Board for the Protection of Human Subjects (Rush IRB), were provided for review at the study information sessions and the study was approved by the Rush IRB as a central review board for all 5 sites. Additionally, informed consent was obtained digitally before the assessment during the mandated research pause.

### Data collection

Baseline data were collected from October 16, 2019, through March 12, 2020. Data relevant to the effects of COVID-19 on study participants during the mandated research pause were collected from June 4, 2020, through July 28, 2020 (Table 1). Socioeconomic and demographic data (sex, marital status, race and ethnicity, education, employment status, and economic stress, including availability of healthy foods in neighborhood) and all psychosocial data (eating competence, health-related quality of life, perceived stress, depressive symptoms) were collected via self-report questionnaires administered by research personnel either in person (baseline) or by video call (during the COVID-19 pause). Research assistants were trained to administer surveys and collect physical measures using standardized, tested protocols.

**Table 1:** Data Collected at Baseline and at Follow-Up During a COVID-19–Mandated Pause in Research, Enhanced Lifestyle for Metabolic Syndrome Trial, US

Characteristic	Baseline (October 16, 2019–March 12, 2020)	Follow-up (June 4, 2020–July 28, 2020)
Socioeconomic or demographic	x	
Anthropometric (height, weight, waist circumference)	x	
Bioclinical (blood pressure, fasting glucose, lipid panel)	x	
**Psychosocial**		
Satter Eating Competence Inventory^a^	x	
36-Item Short Form Health Survey^b^	x	
Perceived Stress Scale^c^	x	x
Patient Health Questionnaire-8^d^	x	x
10-Item US Adult Food Security Module^e^	x	x

a An intra-individual approach to eating and food behaviors that is associated with positive health outcomes, measured on a 16-item scale (7–11).

b Assesses health-related quality of life across 8 categories (12).

c A 14-item instrument that measures the degree to which situations in a person’s life are deemed stressful (13).

d An 8-item self-report measure to diagnose depressive disorders and assess level of severity (14).

e Developed by the US Department of Agriculture (15).

#### Anthropometric data collection

Research assistants measured waist circumference, height, and weight. Body mass index (BMI) was calculated as weight in kilograms divided by height in meters squared (kg/m^2^) and classified as overweight (BMI 25.0–29.9) and obese (BMI ≥30.0).

#### Bioclinical assessment of MetS components

Serum glucose, glycated hemoglobin A_1c_ (HbA_1c_), and lipid panel (HDL cholesterol, total cholesterol, low-density lipoprotein cholesterol, and triglycerides) were determined from a 12-hour fasting blood draw conducted by using standard procedures by Quest Diagnostics (16). Blood pressure was measured with an Omron HEM-907XL (Omron Healthcare, Inc) digital blood pressure monitor following a standard procedure.

#### Psychosocial assessment

**Satter Eating Competence Inventory**. Eating competence is an intra-individual approach to eating and food behaviors that is associated with positive health outcomes, including being more physically active, having better sleep and dietary quality, and having less stress and emotional or disordered eating patterns (7). Eating competence was measured with the 16-item Satter Eating Competence Inventory, which has been validated in samples of men, women, and adolescents in a general population in the US, Finland, Brazil, and Taiwan (7–11). Total scores may range from 0 to 48. Eating competence is defined as a score of 32 or more. Possible ranges vary for the 4 subscales (from 0 to 6 for internal regulation, 0 to 9 for food acceptance, 0 to 15 for contextual skills, and 0 to 18 for eating attitudes and behavior [17]). Cronbach α for the results of the baseline inventory was 0.82.

**36-Item Short Form Health Survey (SF-36)**. The SF-36 assesses health-related quality of life across 8 categories (12). Each category is scored from 0 (poor health) to 100 (very good health) (18). Categories used were the 5-item SF-Mental Health, which assesses general mental health, especially depression and anxiety, and the 4-item SF-Vitality, which assesses energy and fatigue status (19). The SF-36 has been validated for use among people with morbid obesity and the mental health and vitality subscales have been identified as relevant measures in the obese population (20).

**Perceived stress.** The Perceived Stress Scale measures the degree to which situations in a person’s life are deemed stressful (13). With adequate validity and reliability, this instrument can be used to examine the role of stress levels in the etiology of diseases, such as MetS. It consists of 14 items; total scores range from 0 to 56 and can be categorized as low stress (score 0–19) or high stress (score 20–56). Cronbach α was 0.84 at both baseline and follow-up.

**Patient Health Questionnaire-8 (PHQ-8)**. The PHQ-8 is a well-established, validated 8-item self-report measure to diagnose depressive disorders and assess level of severity (14). PHQ-8 scores can range from 0 to 24, with scores of 0 to 4 indicating no to minimal depression, 5 to 9 indicating mild depression, 10 to 14 indicating moderate depression, 15 to 19 indicating moderately severe depression, and 20 to 24 indicating severe depression. Cronbach α was 0.65 at baseline and 0.75 at follow-up.

**Food security.** Food security was measured with the validated US Department of Agriculture’s 10-item US Adult Food Security Module (15). Scores may range from 0 to 10 and are categorized as high food security (score, 0), marginal food security (score, 1 or 2), low food security (score, 3 to 5), and very low food security (score, 6 to 10). Categories of high and marginal food security are considered food secure, and categories of low or very low food security are considered food insecure.

### Data analysis

Each instrument was scored, summed, or categorized as directed. Based on sample homogeneity, race and ethnicity was grouped as non-Hispanic White (hereinafter, White) or racial or ethnic minority (American Indian or Alaska Native; Asian; Black or African American; Hispanic, Latino, Spanish origin; multiracial or multiethnic; or other), and food security was denoted as either food secure or food insecure. Statistical analyses were performed with SPSS Statistics version 28 (IBM Corp). Internal consistency of scales was assessed with Cronbach α. Skewness, kurtosis, and Q–Q plots were examined to determine normality; values between +1 and −1 were considered to indicate a normal distribution. PHQ-8 scores were log10 +1 transformed to achieve normality for the purposes of statistical analyses. Reported means and standard deviations align with the survey response options. PHQ-8 item changes from “not at all” (score of 0) or “several days” (score of 1) to “more than half the days” (score of 2) or “nearly every day” (score of 3) were examined and tallied, especially for movement into the latter 2 categories. Changes from baseline to follow-up were analyzed with paired samples *t* tests, groups were compared with independent *t* tests, and associations were assessed with Pearson *r* (continuous values) and χ^2^ test, or Fisher–Freeman−Halton exact test (categorical values) when cell sizes were less than 5. A general linear model with repeated measures was used to examine change with control for confounding relationships. Wilcoxon and Mann–Whitney *U* tests were used to compare participants with worsened baseline food security with others because of a low sample size in 1 group. Significance was set at *P* < .05.

## Results

Of the 150 enrolled participants, 132 completed both baseline and follow-up assessments. Most (67%) were female, married (55%), White (70%), college-educated (62%), and employed full-time (61%) (Table 2). The mean (SD) age was 57.0 (11.0) years; 44% were aged at least 60 years. Median household income was $86,000 and ranged from $11,000 to $300,000. Most (83%) participants were food secure and reported living in an environment that provided access to high quality fruits and vegetables and low-fat foods. Laboratory and anthropometric measures affirmed a MetS diagnosis. BMI ranged from 24.0 to 61.2 with a mean (SD) of 36.6 (6.9); 84% (n = 111) were classified as obese, 15% (n = 20) as overweight, 1 participant as normal weight. 

**Table 2:** Description of Participants (N = 132) at Baseline, Enhanced Lifestyle for Metabolic Syndrome Trial, US, October 16, 2019–March 12, 2020

Characteristic	No. (%)^a^
**Sex**	
Female	88 (67)
Male	44 (33)
**Marital status**	
Single	30 (23)
Live with partner	4 (3)
Live with spouse	73 (55)
Divorced	16 (12)
Widowed	9 (7)
**Race and ethnicity^b^**	
American Indian or Alaska Native	1 (1)
Asian	3 (2)
Black or African American	26 (20)
Hispanic, Latino, Spanish origin	15 (11)
White	92 (70)
Multiracial or multiethnic	1 (1)
Other	8 (6)
**Education**	
Less than high school	2 (2)
High school	16 (12)
Vocational training	2 (2)
Some college or associate degree	31 (24)
4-year degree	47 (36)
Master’s degree	30 (23)
Doctoral degree	4 (3)
**Employment status**	
Full-time	80 (61)
Part-time	13 (10)
Not working but want to	5 (4)
Chooses to not work	5 (4)
Retired	28 (21)
Employed student	1 (1)
**Paying for basics^c^**	
Very hard	0
Somewhat hard	12 (9)
Not hard at all	120 (91)
**Food security^d^**	
High	109 (83)
Marginal	16 (12)
Low or very low	7 (5)
**Neighborhood food**	
Agree or strongly agree that the fruits and vegetables in their neighborhood are of high quality	123 (93)
Agree or strongly agree that a large selection of fruits and vegetables are available in their neighborhood	127 (96)
Agree or strongly agree that a large selection of low-fat products is available in their neighborhood	123 (93)
**Eating competent^e^**	62 (47)
**Eating competence scores, mean (SD) [median; range]**	
Eating competence^e^	30.7 (7.2) [31.0; 10–47]
Eating attitudes^f^	13.8 (3.1) [13.0; 4–18]
Food acceptance^g^	4.7 (1.9) [5.0; 0–9]
Internal regulation^h^	4.0 (1.4) [4.0; 0–6]
Contextual skills^i^	8.8 (3.0) [9.0; 1–15]

a All values are number (percentage) unless indicated. Percentages may not sum to 100 because of rounding.

b n = 131, 1 person refused to answer; ≥1 selection was possible.

c Question was, “How hard is it for you to pay for the very basics like food, housing, medical care, and heating?” Options were very hard, somewhat hard, not hard at all.

d Assessed by the 10-Item US Adult Food Security Module (15). Scores range from 0 to 10; food secure = score of 0; marginal = score of 1 or 2; low or very low = score 3–10.

e Determined by the Satter Eating Competence Inventory. Eating competence is an intra-individual approach to eating and food behaviors that is associated with positive health outcomes, measured on a 16-item scale (7–11). Scores may range from 0 to 48; eating competence is defined as a score ≥32.

f Possible range 0–18.

g Possible range 0–9.

h Possible range 0–6.

i Possible range 0–15.

### Baseline characteristics

Eating competence status (ie, score of <32 vs score of ≥32) was not associated with race or education level. Competent eaters (n = 62) were more likely to have high food security than noncompetent eaters (n = 70) (92% vs 74%; *P* = .008). Diastolic and systolic blood pressure, total cholesterol, HDL cholesterol, triglycerides, serum glucose, and HbA_1c_ did not differ by eating competence status. However, competent eaters had a lower BMI, had greater vitality, and perceived less stress than noncompetent eaters (Table 3). Additionally, we found fewer mean (SD) depressive symptoms among competent eaters (1.6 [2.0]) than among noncompetent eaters (2.5 [2.5]) (*P* = .04).

**Table 3:** Comparison of Selected Baseline Characteristics of Eating-Competent and Non–Eating-Competent Participants, Enhanced Lifestyle for Metabolic Syndrome Trial, US, October 16, 2019–March 12, 2020

Characteristic	Eating Competent^a^ (n = 62)	Non–Eating Competent^a^ (n = 70)	*P* value^b^
Body mass index^c^	35.3 (6.4)	37.7 (7.1)	.049
Age, y	59.2 (10.5)	55.1 (11.1)	.03
Vitality^d^	63.6 (18.0)	55.0 (19.8)	.01
Mental health^e^	84.2 (8.7)	78.4 (12.3)	.002
Patient Health Questionnaire-8^f^	1.6 (2.0)	2.5 (2.5)	.04
Perceived stress^g^	17.0 (5.8)	19.9 (6.6)	.007
Systolic blood pressure, mm Hg	131.6 (16.6)	129.6 (15.9)	.48
Diastolic blood pressure, mm Hg	81.0 (11.1)	84.3 (10.3)	.08
Triglycerides, mg/dL	170.0 (79.6)	177.3 (135.2)	.71
High-density lipid cholesterol, mg/dL	46.8 (10.1)	45.8 (11.3)	.59
Total cholesterol, mg/dL	193.2 (44.1)	192.9 (42.3)	.97
Serum glucose, mg/dL	98.4 (13.0)	97.4 (10.8)	.61
Hemoglobin A_1c_	5.8 (0.3)	5.8 (0.3)	.65
Food secure, %	92	74	.008

a Determined by the Satter Eating Competence Inventory. Eating competence is an intra-individual approach to eating and food behaviors that is associated with positive health outcomes, measured on a 16-item scale (7–11). Scores may range from 0 to 48; eating competence is defined as a score ≥32. Values are mean (SD) unless otherwise indicated.

b Determined by independent *t* test; *P* < .05 considered significant.

c Calculated as weight in kilograms divided by height in meters squared (kg/m^2^); obesity defined as body mass index ≥30.0.

d Measured by the 4-item SF-Vitality, which assesses energy and fatigue status, and is part of the 36-Item Short Form Health Survey, which assesses health-related quality of life across 8 categories (12). Scores range from 0 to 100; higher scores indicate greater vitality.

e Measured by the 5-item SF-Mental Health, which assesses general mental health, especially depression and anxiety, and is part of the 36-Item Short Form Health Survey, which assesses health-related quality of life across 8 categories (12). Scores range from 0 to 100; higher scores indicate better mental health.

f 8-Item self-report measure to diagnose depressive disorders and assess level of severity (14). Scores can range from 0 to 24: 0–4, no to minimal depression; 5–9, mild; 10–14 moderate; 15–19, moderately severe; and 20–24, severe.

g Assessed by the 14-item Perceived Stress Scale, which measures the degree to which situations in a person’s life are deemed stressful (13). Total scores range from 0 to 56 and can be categorized as low stress (score 0–19) or high stress (score 20–56).

The SF-36 revealed adequate vitality and mental health: mean (SD) subscale scores were 59 (19.4; range, 6–94) for vitality and 81.1 (11.1; range, 40–100) for mental health.

Baseline PHQ-8 values ranged from 0 to 9 (minimal to mild depression) with a median of 1.0 and were not normally distributed. After the log10 +1 transformation, we found greater frequency of depressive symptoms among women (n = 88; mean, 2.4; SD, 2.4) than among men (n = 44; mean, 1.4; SD, 2.1]) (*P* = .02). PHQ-8 scores were not associated with age or income but were positively related to BMI (*r* = 0.22; *P* = .01) and perceived stress (*r* = 0.40; *P* < .001). In addition, greater frequency of depressive symptoms was associated with poorer mental health (*r* = −0.49, *P* < .001), less vitality (*r* = −0.60, *P* < .001), and lower levels of eating competence (*r* = −0.22, *P* = .01).

At baseline, perceived stress scores did not differ between men and women, college-educated versus non–college-educated participants, or White versus racial and ethnic minority participants, but they were higher among noncompetent eaters (mean [SD] score, 19.9 [6.6]) than among competent eaters (mean [SD] score, 17.0 [5.8]) (*P* = .007). Perceived stress scores were associated with worse mental health (*r* = −0.71, *P* < .001), lower vitality (*r* = −0.5, *P* < .001), and lower levels of eating competence (*r* = −0.31, *P* < .001).

Food security (mean [SD] score, 0.44 [1.3]) was associated with older age (*r* = −0.24, *P* = .005), greater household income (*r* = −0.20, *P* = .03), better mental health (*r* = −0.19, *P* = .03), less stress (*r* = 0.24, *P* = .006), and eating competence (total score −0.24, *P* = .005). Mean (SD) food security scores were higher among White participants than racial or ethnic minority participants (0.26 [1.0] vs 0.85 [1.7]; *P* = 0.003).

### Changes from baseline to follow-up

#### Depressive symptoms

Mean (SD) frequency of depressive symptoms significantly increased by 1.4 (3.0) points with a median change of 1 point; changes ranged from a decrease of 7 points to an increase of 10 points; at follow-up 74 (56%) participants had a higher PHQ-8 score than at baseline, with 31 (23%) participants increasing by 4 points and 14 (11%) increasing by 6 or more points. To address a concern about possible confounding between treatment before the COVID-19–mandated pause and follow-up responses as an explanation for the increases in depressive symptoms, we conducted additional analyses that excluded data from the 2 sites that had some treatment during this time (ie, mailings/calls to the self-directed arm and the 1 site with 2 group-based pre-pause sessions). The increase in PHQ-8 scores remained significant at *P* < .001 with a similar change of 1.6 (SD, 3.0) for the remaining 3 sites (n = 70). Change in depressive symptoms was not related to baseline values for age, BMI, income, vitality, eating competence, or perceived stress. PHQ-8 change from baseline to COVID-19–mandated pause remained significant when we controlled for age (*P* = .02), sex (*P* < .001), and eating competence (*P* < .001) but not when we controlled for baseline BMI, vitality, or perceived stress. At baseline, as required for inclusion, no participant had a PHQ-8 score greater than 9. However, at follow-up, 10 (8%) participants had a score of 10 or more (3 had a score of 10, five scored 11, and 1 each scored 13 and 14), indicating clinically moderate presence of depressive symptoms. These 10 participants were not unique in sociodemographic characteristics; however, compared with those who scored in the normal range, they had lower mean (SD) baseline vitality scores (60.3 [18.9] vs 44.4 [20.7]; *P* = .01) and lower mean (SD) baseline mental health scores (82.0 [10.4] vs 71.0 [14.3]; *P* = .002). Therefore, these 10 participants may have been more susceptible to developing and increasing the frequency of depressive symptoms during the early stages of the COVID-19 pandemic.

Examination of PHQ-8 individual items showed 41 participants with movement of at least 1 item into a category of concern (ie, a score of 2 or 3). All sites had at least 3 such participants, with 1 site having 7, another site 9, and 2 sites each having 11 participants. Eight participants from 3 separate sites had movement of 2 PHQ-8 items into a category of concern, 9 participants from 4 separate sites showed this pattern of movement for 3 PHQ-8 items, and 1 site had 2 participants show this pattern for 4 items each. Thirteen participants (representing all 5 sites) reported worsening for the first or second (or both) PHQ-8 items.

#### Perceived stress

Mean (SD) perceived stress scores significantly decreased from baseline (−3.7 [6.5]), with change ranging from −20 to +20 points. Increased perception of stress was associated with increased depressive symptoms (*r* = 0.33, *P* < .001). Compared with those with unchanged or less stress at follow-up (n = 93), participants with greater stress (n = 39) at follow-up had significantly greater mean (SD) increases in depressive symptoms (1.0 [2.7] vs 2.4 [3.4]; *P* = .02). Change in stress from baseline to follow-up remained significant when we controlled for baseline eating competence status (*P* < .001), baseline mental health (*P* = .03), and PHQ-8 score (*P* < .001), and it became a trend when we controlled for vitality (*P* = .05).

#### Food security

Fewer participants were food insecure or marginally food secure at follow-up than at baseline (13 vs 23, respectively) (Table 4). Changes in food security status were most pronounced among participants with marginal food security. Of the 132 participants, 103 (78%) had high food security at both baseline and follow-up and only 1 participant (<1%) had low or very low food security throughout. However, of the 16 participants with marginal food security at baseline, 13 (81%) moved to a high food security status at follow-up. Of the 7 participants who had low or very low food insecurity at baseline, 3 migrated to a high food security status and 3 migrated to marginal food security.

**Table 4:** Change from Baseline to Follow-Up Among Participants During a COVID-19–Mandated Pause in Research, Enhanced Lifestyle for Metabolic Syndrome Trial, US

Factor	Baseline (October 16, 2019–March 12, 2020)	Follow-up (June 4, 2020–July 28, 2020)	*P* value
**Mental health, mean (SD)**			
Patient Health Questionnaire-8, mean (SD) score^a^	2.1 (2.3)	3.5 (3.4)	<.001^b^
Perceived Stress Scale, mean (SD) score^c^	18.5 (6.4)	14.9 (7.2)	<.001^b^
**Food security,^d^ no. (%)**			
High	109 (83)	119 (90)	<.001^e^
Marginal	16 (12)	12 (9)	
Low or very low	7 (5)	1 (1)	

a 8-Item self-report measure to diagnose depressive disorders and assess level of severity (14). Scores can range from 0 to 24: 0–4, no to minimal depression; 5–9, mild; 10–14 moderate; 15–19, moderately severe; and 20–24, severe.

b Paired-samples *t* test.

c Assessed by the 14-item Perceived Stress Scale, which measures the degree to which situations in a person’s life are deemed stressful (13). Total scores range from 0 to 56 and can be categorized as low stress (score 0–19) or high stress (score 20–56).

d Measured by the 10-Item US Adult Food Security Module, developed by the US Department of Agriculture (15).

e Fisher–Freeman–Halton exact test.

In an examination of changes in food security and psychosocial differences, we observed that participants who remained in the marginal, low, or very low food security category from baseline to follow-up were more likely to be in a racial or ethnic minority group, found it hard to pay for basics, were less likely to be competent eaters, were younger, and perceived more stress (Table 5). However, participants who moved from the marginal, low, or very low food security category to the high food security status still found it hard to pay for basics, were less likely to be competent eaters, had lower scores for contextual skills, and were the youngest participants.

**Table 5:** Characteristics of Participants in the Enhanced Lifestyle for Metabolic Syndrome Trial, According to Movement Between High Food Security and Marginal, Low, or Very Low Food Security From Baseline to Follow-Up During a COVID-19–Mandated Pause in Research, US^a^

Characteristic^b^	High food security to high food security (n = 103)	Marginal, low, very low food security to high food security (n = 16)	High food security to marginal, low, very low food security (n = 6)	Marginal, low, very low food security to marginal, low, very low food security (n = 7)	*P* value^c^
**Sociodemographic, no. (%)**					
White race (vs all other categories of race and ethnicity)	78 (76)	8 (50)	4 (67)	2 (29)	.02
4-Year degree or more (vs <4-year degree)	65 (63)	12 (75)	2 (33)	2 (29)	.09
Male vs female	34 (33)	3 (19)	3 (50)	4 (57)	.25
Employed full-time (vs all other categories of employment)	61 (59)	11 (69)	3 (50)	5 (71)	.76
Somewhat hard to pay for basics (vs not hard at all)	4 (4)	4 (25)	1 (17)	3 (43)	<.001
Eating competent (vs non–eating competent)^d^	55 (53)	3 (19)	2 (33)	2 (29)	.03
Low or very low food security (vs high or marginal)^e^	0	3 (19)	0	4 (57)	<.001
Follow up PHQ-8 score ≥10 (vs <10) f	6 (6)	1 (6)	1 (17)	2 (29)	.13
**Scores and scales, mean (SD)**					
EC score^d^^,^ g	31.9 (6.6)	27.0 (6.3)	26.3 (7.5)	24.4 (9.9)	.001
EC–eating attitudes^h^	13.6 (2.9)	10.9 (3.7)	11.7 (2.7)	12.1 (3.5)	.006
EC–food acceptance^i^	5.0 (1.8)	4.6 (1.7)	2.5 (1.6)	3.0 (2.8)	.001
EC–internal regulation^j^	4.1 (1.4)	3.8 (1.3)	4.2 (1.3)	3.0 (2.1)	.20
EC–contextual skills^k^	9.2 (2.9)	7.8 (2.2)	8.0 (4.2)	6.3 (3.1)	.02
Annual household income, $	101,733 (59,769)	84,615 (58,703)	86,067 (52,283)	65,714 (63,458)	.35
Body mass index^l^	36.2 (7.0)	37.2 (5.1)	37.4 (8.4)	39.6 (7.1)	.80
Age, y	58.3 (10.1)	40.4 (13.5)	62.8 (6.1)	47.7 (10.7)	.002
Perceived stress score^m^	18.3 (6.1)	18.2 (5.8)	15.0 (7.8)	25.3 (7.4)	.02
Short form-Vitality^n^	59.8 (19.5)	60.6 (16.7)	55.2 (21.1)	47.3 (21.3)	.39
Short form-Mental Health^o^	81.9 (10.8)	79.1 (10.0)	81.7 (14.0)	73.6 (14.4)	.23
PHQ-8^f^^,^ p	2.1 (2.4)	2.3 (2.4)	1.5 (1.4)	2.6 (2.2)	.86

Abbreviations: EC, eating competence; PHQ-8, Patient Health Questionnaire-8.

a Baseline took place October 16, 2019–March 12, 2020; follow-up took place June 4, 2020–July 28, 2020.

b All characteristics were noted at baseline unless otherwise indicated.

c Fisher–Freeman–Halton exact test used to determine *P* values for number (percentage); analysis of variance used to determine *P* values for mean (SD).

d Determined by the Satter Eating Competence Inventory. Eating competence is an intra-individual approach to eating and food behaviors that is associated with positive health outcomes, measured on a 16-item scale (7–11). Scores may range from 0 to 48; eating competence is defined as a score ≥32.

e Assessed by the 10-Item US Adult Food Security Module (15). Scores range from 0 to 10; food secure = score of 0; marginal = score of 1 or 2; low or very low = score 3–10.

f 8-Item self-report measure to diagnose depressive disorders and assess level of severity (14). Scores can range from 0 to 24: 0–4, no to minimal depression; 5–9, mild; 10–14 moderate; 15–19, moderately severe; and 20–24, severe.

g Score remained significantly different among food security movement groups when we controlled for annual household income (*P* = .001) and difficulty paying for basics (*P* = .046).

h Possible range 0–18. Significant difference found between high food security to high security group and marginal, low, very low food security to high food security group.

i Possible range 0–9.

j Possible range 0–6.

k Possible range 0–15.

l Calculated as weight in kilograms divided by height in meters squared (kg/m^2^); obesity defined as body mass index ≥30.0.

m Assessed by the 14-item Perceived Stress Scale, which measures the degree to which situations in a person’s life are deemed stressful (13). Total scores range from 0 to 56 and can be categorized as low stress (score 0–19) or high stress (score 20–56). Those continuing to be marginally, low, or very low food secure perceived significantly more stress than those continuing to be highly food secure or moving from high to marginal, low, or very low food security.

n 4-Item SF-Vitality assesses energy and fatigue status, and is part of the 36-Item Short Form Health Survey, which assesses health-related quality of life across 8 categories (12). Scores range from 0 to 100; higher scores indicate greater vitality.

o 5-Item SF-Mental Health assesses general mental health, especially depression and anxiety, and is part of the 36-Item Short Form Health Survey, which assesses health-related quality of life across 8 categories (12). Scores range from 0 to 100; higher scores indicate better mental health.

p Analysis of variance with transformed PHQ-8 scores.

The 6 participants who moved from baseline high food security to marginal food security had no unique bioclinical, psychosocial, or sociodemographic characteristics.

## Discussion

People diagnosed with MetS and participating in a lifestyle intervention to reverse MetS did not experience increased food insecurity or perceive increased stress during the COVID-19 pandemic despite being considered at high risk for SARS-CoV-2 infection (21,22). In contrast, food insecurity increased among the general US population during the pandemic (1), especially among racial and ethnic minority populations and in households with job disruptions (23,24). Two studies, one by Hossain et al (25) and another by Kshirsagar et al (26), found increases in stress in their study populations, especially among women and people with MetS-related comorbidities. The socioeconomic status of participants in our sample, characterized by a high percentage of people with a college degree and a comfortable income level and a small percentage of racial and ethnic minority participants, likely explains the discrepant findings. Improved dietary quality during the pandemic has been reported in studies involving samples with similar socioeconomic demographic characteristics (27,28). Government-driven pandemic responses (eg, pauses in rent or energy payments, increases in unemployment benefits) may have affected food security in our study, especially in moving people from marginal to high food security.

In contrast to our results on food security and stress, we found increases in depressive symptoms; 31 (23%) participants in our study had a PHQ-8 score that was 4 points higher at follow-up than at baseline. In comparison, 14% of a racially diverse US sample (n = 290) and 20% of the 84 non-Hispanic White participants in that sample had continued or worsening depressive symptomatology (29). Missing friends, family, and favorite or special events; limited agency and free will; and the uncertainty of COVID-19 transmission and treatment amid abject misinformation and speculation (2) are plausible explanations for the increases in depressive symptoms. Our sample was more educated and reported employment in sectors that transitioned to working from home during the pandemic, meaning more time to plan menus, cook, and consider food resource management. We speculate that less commuting among those who could work at home during the pandemic with the gain in personal time and less interaction with the worksite could explain the lower perceived stress among our sample. However, our study participants were queried only about perceived stress during the past month, not about pandemic-related stress. 

### Strengths and limitations

Strengths of this study are its rigorous, clinical inclusion criteria affirming a MetS diagnosis, the use of validated instruments administered by trained research personnel, and a geographically diverse sample. The sample appears to be representative of the MetS population in the US with respect to age, education, and sex (30). Generalization to the Hispanic population may be limited. MetS prevalence is increasing among the Hispanic population (30), and only 11% of our sample was Hispanic and 6% self-identified as multiethnic or “other” race that may include people who are Hispanic or Latino. In addition, all follow-up data were collected after several months of the research pause and lifestyle disruption. Conclusions are limited by the lack of follow-up anthropometric measures to assess changes in BMI and waist circumference. However, public health mandates made this type of measurement impossible. Additionally, analyses could not control for COVID-19 experiences (eg, losing a loved one or requiring hospitalization) because research and institutional protocols prohibited asking if a participant had contracted COVID-19 or had been quarantined. A study limitation is that all 5 sites were not at the same stage at the time of the research pause. Two sites had randomized participants into each study arm; 3 sites had not, and among participants randomized (n = 61), 31 enrolled in the self-directed study arm at the time of the pause continued to receive mailings with a check-in telephone call during the pause. Participants randomized to the group-based arm received check-in calls; nonrandomized sites had little or no contact. However, removal of the site that had started the group-based arm and continued the self-directed arm did not alter our finding of increased depressive symptoms at follow-up. Furthermore, all sites reported participants with increased depressive symptoms, and changes among 19 participants, representing all sites, were compatible with a clinical concern. Finally, Shader (31) noted that increases in depressive symptoms during the pandemic may indicate demoralization, not depression. Demoralized people benefit from lifestyle interventions that include encouragement and support, especially activities leading to a sense of mastery. Thus, the increase in depressive symptoms may have been temporary, abating with the start of the ELM intervention.

### Conclusion

Our findings have implications for future assessment of the ELM intervention. There is assurance that any postintervention changes in stress and food security in our first cohort were not caused by the COVID-19–related research pause. For this cohort, changes in depressive symptoms from baseline to follow-up can be examined by considering PHQ-8 values during the research pause, minimizing the threat of history to internal validity. In addition, knowing that PHQ-8 values can worsen in the absence of the intervention will be useful when interpreting outcome data.
